# Spina Bifida Successfully Treated by Ligature and Puncture

**Published:** 1858-12

**Authors:** J. G. Wilson

**Affiliations:** Fellow of the Faculty of Physicians and Surgeons, Physician to the Glasgow Lying-in Hospital and Dispensary, etc.


*Spina Bifida Successfully Treated by Ligature and Puncture.* By J. G. WILSON, M. D. Fellow of the Faculty of Physicians and Surgeons, Physician to the Glasgow Lying-in Hospital and Dispensary, etc.

The following case of spina bifida occurred in the practice of my late father, the report of which, although not so full or minute as might have been desired, is extracted from his case book:—

"About the middle of July last [year not stated] a male infant was brought to me a few days after birth with spina bifida over the lumbar region. The tumor was about the size of a small orange, and rose by a neck nearly an inch in diameter from the middle of the lumbar vertebræ. It was distinctly fluctuating, irregular, and nodulated on the surface, and covered by thin, delicate and transparent membrane. The child appeared to suffer much pain and uneasiness when pressure was applied to the tumor, and required to be constantly laid on its sides or abdomen. The power of voluntary motion in the lower limbs was little if at all impaired.

"What I considered a favorable circumstance in this case was, that the pedicle or neck of the tumor was covered with the ordinary integument; and this led me to attempt its removal by ligature after consulting with some of my medical friends. A ligature was applied not very tightly at first, and a new one put on every day. The tumor enlarged considerably, and it was frequently punctured with a fine needle. A large quantity of clear serous fluid exuded from the puncture, and it was not till the fourteenth day after the first application of the ligature that the sac began to shrink and shrivel up. The membranous covering became reddish, and ultimately black, and came away on the eighteenth day after application of the first ligature, leaving a rew tender surface about a quarter of an inch in extent. This small sore, by the use of simple water-dressing, combined with pressure, gradually healed, and a firm cicatrix was formed in the space of three weeks. Before the closure of this sore a slight fissure or defect in the canal through which the tumor protruded was distinctly felt. The infant enjoyed excellent health during the whole time. It complained a little each time the ligature was tightened, and appeared on the whole, less troublesome than formerly. The child was brought to me three months afterwards for vaccination, and was in a healthy, thriving condition, with the back perfectly sound."—*London Medical Times and Gazette.*

